# Inhibition of Osteoclast Differentiation and Bone Resorption by Bisphosphonate-conjugated Gold Nanoparticles

**DOI:** 10.1038/srep27336

**Published:** 2016-06-02

**Authors:** Donghyun Lee, Dong Nyoung Heo, Han-Jun Kim, Wan-Kyu Ko, Sang Jin Lee, Min Heo, Jae Beum Bang, Jung Bok Lee, Deok-Sang Hwang, Sun Hee Do, Il Keun Kwon

**Affiliations:** 1 Kyung Hee University, Department of Dentistry, Graduate School, 26, Kyungheedae-ro, Dongdaemun-gu, Seoul 02477, Korea; 2 The George Washington University, Department of Mechanical and Aerospace Engineering, Washington DC 20052, United States; 3 Konkuk University, Department of Clinical Pathology, College of Veterinary Medicine, Seoul 05029, Korea; 4 Kyung Hee University, Department of Dental Education, School of Dentistry, 26, Kyungheedae-ro, Dongdaemun-gu, Seoul 02477, Korea; 5 Vanderbilt University, Department of Biomedical Engineering, Nashville, TN 37235, United States; 6 Department of Korean Gynecology, Conmaul Hospital, Kyung Hee University, Seoul 02477, Korea; 7 Kyung Hee University, Department of Dental Materials, School of Dentistry, 26, Kyungheedae-ro, Dongdaemun-gu, Seoul 02477, Korea

## Abstract

In recent years, gold nanoparticles (GNPs) have been reported to affect the regeneration of bone tissue. The goal of this study was to improve bone tissue regeneration by using targeted GNPs. We fabricated a functionalized GNPs conjugated with alendronate (ALD), of the bisphosphonate group. Subsequently, the ALD, GNPs, and ALD conjugated GNPs (GNPs-ALD) were analyzed by ultraviolet-visible absorbance (UV-vis) spectrophotometer, Attenuated total reflectance Fourier transform infrared spectrometer (ATR-FTIR), and thermo gravimetric analysis (TGA). The prepared GNPs-ALD were used to investigate their inhibitory effects on the receptor activator of nuclear factor- κb ligand (RANKL)-induced osteoclastogenesis in bone marrow-derived macrophages (BMMs). Additionally, the GNPs-ALD were applied to ovariectomy (OVX)-induced osteoporotic mice and the experiments were evaluated. ALD was found to be successfully conjugated to the GNPs surface, and it displayed significant adhesion onto the bone surface. The *in-vitro* study indicated that the GNPs, ALD and GNPs-ALD suppressed osteoclast formation in a dose-dependent manner. Furthermore, in the OVX mouse model, the mice treated GNPs-ALD had higher bone density as compared to other OVX mice groups. The results from these tests indicated that GNPs-ALD can be useful agents for preventing and treating osteoporosis.

Osteoporosis (OP) is a skeletal disease characterized by low bone mass and micro architectural deterioration of bone tissue. This disease is especially prevalent in menopausal women^1^. Recently, OP has become a major public health problem all around the world. This is due to increased life expectancy with the advancement of medical technology. Because of this, the number of women experiencing menopause is increasing rapidly, and the frequency of OP is increasing too. Interest in prevention and treatment of OP has been rising along with the high incidence of disease. There have been various *in vitro* and *in vivo* experiments reported for OP treatment^2^,^3^,^4^,^5^.

Bisphosphonates have been used for treatment of metastatic bone disease, Paget’s disease, multiple myeloma, hypercalcemia of malignancy, and human breast carcinoma^6^,^7^,^8^. These drugs are commonly used to decrease bone adsorption by inhibiting the activity of osteoclasts. This inhibition is due to many effects such as cytoskeletal disruption, changing intracellular protein traffic, blocking intracellular signal transduction pathways, and induction of osteoclast apoptosis^9^,^10^.

By doing this, bisphosphonates suppress bone-resorption. Amongst bisphosphonates, 4-amino-l-hydroxybutylidene-l, l-bisphosphonate (alendronate, ALD), has been widely used to reduce bone fracture and to cause a continued increase in bone mineral density in postmenopausal osteoporotic patients^11^,^12^. However, side effects occur when the drug is used long-term at high doses. The excessive inhibition of bone resorption may cause problems such as suppression of bone formation and jaw necrosis^13^,^14^. This is due to difficulty with repairing small bone damage as the bone conversion is inhibited. This is especially prevalent with alveolar bone, which has a ratio of bone conversion 3–10 times higher than other bone tissue in the body. The oral mucosa, which is weaker and thinner than mucosa of other regions, can be negatively affected by the drugs^15^,^16^,^17^,^18^. To avoid these effects, it is necessary to have a drug-delivery system using a carrier to target specific sites.

Nanoparticles, commonly considered as having a diameter of particles of 100 nm or under, show distinctive properties as compared to larger particles or bulk material. Various nanoparticles have shown promise in studies of bone tissue regeneration^19^. Amongst these various nanoparticles, we noticed gold nanoparticles (GNPs) because they are effective for both promoting osteo-differentiation and inhibiting osteoclast formation. Through many studies, GNPs have been established to have some advantages such as acceleration of osteoblast differentiation^20^, inhibitive action of adipose derived stem cell differentiation, suppression of osteoclast formation, and promotion of bone formation in bone tissue regeneration^21^. Heo at al. described that GNPs treatment demonstrated significantly higher bone tissue regeneration in rabbit calvaria tests as compared to control^22^. However, GNPs can lead to toxicity when injected into the body^23^. Thus, it is necessary to modify the surface of these particles so that they target specifically to bone tissue.

In this study, ALD was coupled onto the GNPs surface (GNPs-ALD) in order to facilitate drug delivery for treatment of osteoporosis. The aim of this study was to prepare therapeutic agents based on GNPs loaded with drug, and to demonstrate the synergistic anti-osteoclast differentiation effect of the combined agent in a mouse-based model. We characterized the synthesized GNPs-ALD by measuring intermolecular bonds, ultraviolet wavelengths, thermogravimetry, and other methods. Subsequently, this agent was evaluated for its cytotoxicity and anti-differentiation effects on bone marrow-derived macrophages (BMMs) as compared with GNPs and ALD solutions, separately. Additionally, these conjugated agents were applied to ovariectomy (OVX)–induced osteoporotic mice and the experiments were evaluated.

## Results

### Characterization of GNPs, ALD, and GNPs-ALD

**Figure 1:**
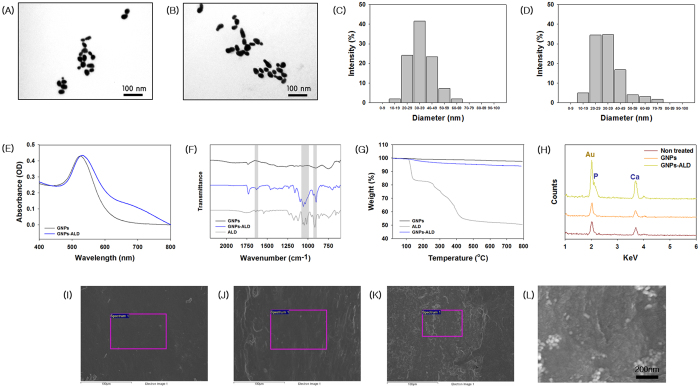
Characterization of GNPs, ALD, and GNPs-ALD. TEM images of the GNPs (**A**) and GNPs-ALD (**B**) and their dynamic light scattering intensities (**C,D**). Ultraviolet-visible spectrophotometer graph of GNPs and GNPs-GNPs (**E**), ATR-FTIR spectrum (**F**) and TGA (**G**) of GNPs, ALD and GNPs-ALD. The tibias of mice were immersed in either blank (control), GNPs, or GNPs-ALD solutions for 24 h. These were measured by using FE-SEM and energy dispersive x-ray spectroscopy (EDX). The results of EDX (**H**) and FE-SEM images of the measured place by EDX (**I**) contained nothing, (**J**) contained GNPs, (**K**) contained GNPs-ALD, (**L**) enlargement of (**K**) at the tibias. Scale bars are 100 nm (**A,B**), 100 μm (**I–K**) and 200 nm (**L**).

The GNPs generated were about 30 nm in diameter and at a concentration of 540 μM. These were conjugated to ALD by adding ALD solution. The shapes of the synthesized particles were visually confirmed as well as the size of about 20–40 nm using a transmission electron microscope (TEM) (Fig. 1A,B). There is no visual difference between the GNPs image (Fig. 1A) and the GNPs-ALD image (Fig. 1B). To clarify the diameter and uniformity of the synthesized particles, dynamic light scattering (DLS) was performed (Fig. 1C,D, Table 1). Both GNPs diameter and GNPs-ALD diameter were found to be mostly distributed in the size range of 20–49 nm (GNPs: 89.06%, GNPs-ALD: 85.94%), and particle size displayed a mean diameter of 34.7 ± 1.8 (GNPs) and 32.9 ± 0.7 (GNPs-ALD). The zeta potential of the GNPs and GNPs-ALD was indicated using a zeta potential analyzer (Table 1). The surface charge of GNPs and GNPs-ALD were 0 and −40.75 ± 2.71 mV, respectively. The ultraviolet-visible absorbance (UV-vis) spectrum of the particles was measured by ultraviolet-spectrophotometer (Fig. 1E). The wavelengths corresponding to the maximum absorbance in GNPs and GNPs-ALD were found at 525 and 531 nm, respectively. In this graph, the shape of the curve appears similar, but slightly different due to a shift in the maximum value. Attenuated total reflectance fourier transform infrared (ATR-FTIR) was also measured and the peaks were shown at 926 cm^−1^, 1020–1050 cm^−1^ and 1650 cm^−1^ in the ATR-FTIR spectra of ALD and GNPs-ALD (Fig. 1F). However, these ALD peaks are not observed in the GNPs. Thermo gravimetric analysis (TGA) analysis showed a decrease of mass by pyrolysis of materials (Fig. 1G). The remaining mass ratio of GNPs, GNPs-ALD, and ALD after heating up to 800 °C were 97.9, 94.24 and 49.99%, respectively. Two sharp decreases are observed in the TGA graph for the ALD.

### Immobilization of GNPs and GNPs-ALD on the bone

The immobilization of synthesized particles on the tibia of mice was evaluated by field emission scanning electron microscopy (FE-SEM) and energy dispersive X-ray spectroscopy (EDX) analysis. Figure 1(I–L) shows the immobilized particles on the surface of bone as images and component ratios after washing. In the image of control (I) and GNPs (J) groups, there is no difference and the surface has little to no nanoparticles bound to it. However, a large amount of particles was observed to be attached to the surface in the image of GNPs-ALD (K and L). Furthermore, the Au peak appeared only in a sample containing particles adhered to the bone using GNPs-ALD (H).

### Endocytosis of GNPs and GNPs-ALD

**Figure 2:**
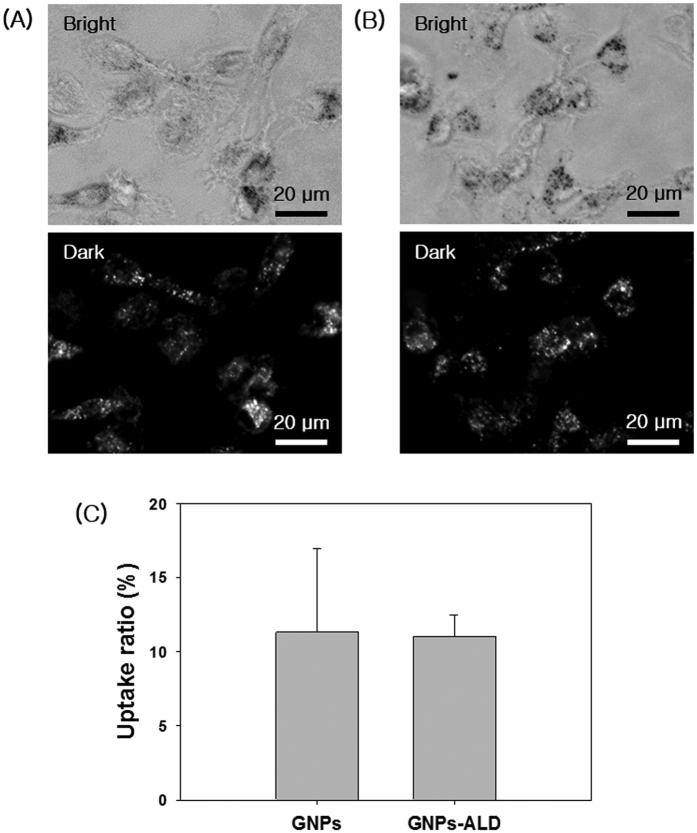
Optical images and dark field images of BMMs incubated with 20 μM GNPs (**A**) and GNPs-ALD (**B**) at 37 °C, 5% CO2 for 12 h. The scattering images calculated by Image J program (bright spot area per total area), and the results are shown as mean ± SD of triplicate experiments (n = 3). Scale bars are 20 μm. Images collected at 400× magnification.

The endocytosis of GNPs and GNPs-ALD in BMMs that had been seeded onto a confocal dish was observed using optical dark field microscopy after treatment with 20 μM of each respective particle solution (Fig. 2A,B). In dark field images, the endocytosed GNPs or GNPs-ALD were displayed as white or bright-gray spots. The uptake ratio was calculated by using Image J program (Fig. 2C). The BMMs and particles were well-dispersed throughout. The white spot ratio of GNPs ALD were 11.33 ± 5.65 and 11.05 ± 1.45%, respectively. In the cell, GNPs tend to aggregate, however the GNPs-ALD were more evenly distributed more than the GNPs.

### Cytotoxicity test of GNPs and ALD

**Figure 3:**
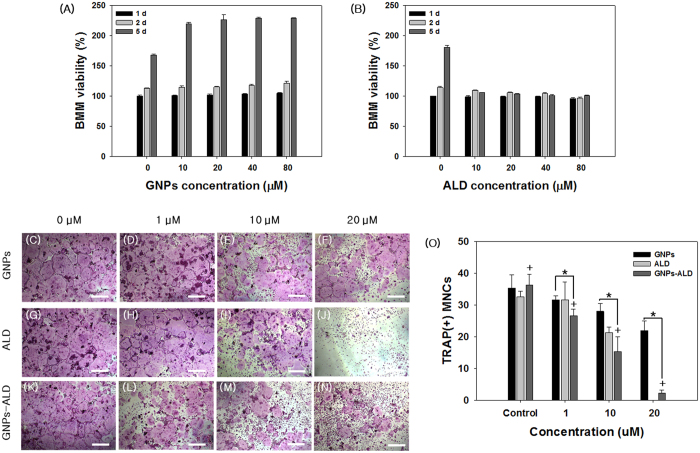
Evaluation of viability of BMMs at various concentrations of GNPs (**A**) and ALD (**B**) for 1 day, 2 days, and 5 days. Results are mean ± SD of triplicate experiments. Tartrate-resistant acid phosphatase (TRAP) staining images of treated GNPs (**C–F**), ALD (**G–J**) and GNPs-ALD (**K–N**) using various concentrations on the osteoclastic differentiated BMMs and the TRAP + MNCs were counted (**O**). Scale bar is 500 μm. Results are mean ± SD of triplicate experiments. A “*” indicates a significant difference of p < 0.05 between two groups, and a “^+^” indicates a significant difference as compared with control group.

The BMMs were exposed to GNPs and ALD and tested using WST-1 assay (Fig. 3A,B). BMMs cultured in medium not containing GNPs and ALD for 24 hours were used as the positive control (100%). The viability of BMMs cultured with 80 μM GNPs for 1 day, 2 days and 5 days were increased by 4.9%, 21.1% and 28.5% over control, respectively (Fig. 3A). In addition, the viability of BMMs was more increased when the GNPs concentration was increased (Supplementary S3A). However, the group treated with various concentrations of ALD showed a marked decrease in cell viability at all doses after 5 days (Fig. 3B). At doses higher than 150 μM, the viability values decreased sharply.

### Evaluation of osteoclast inhibition effect

We also checked the effect of these drug systems to suppress differentiation of BMMs to osteoclast after stimulation with 100 ng/ml receptor activator of nuclear factor-kappa B ligand (RANKL) for 5 days. This was determined using tartrate-resistant acid phosphatase (TRAP) staining assay, and all groups treated with particles and/or drugs showed reduced differentiation to osteoclasts in a dose dependent manner (Fig. 3C–O). The inhibitory effects of GNPs, ALD, and GNPs-ALD against RANKL-stimulated osteoclast differentiation of BMMs were qualitatively evaluated by staining the TRAP+MNCs^24^,^25^. The positive control (no drug) displayed a substantial quantity of TRAP+MNCs, and the 1–20 μM of GNPs and GNPs-ALD inhibited the formation of TRAP+MNCs. The ALD group was found to fully inhibit differentiation of osteoclast at 20 μM, however this suppression did not appear at the lower 1 μM concentration. The number of TRAP+MNCs steadily declined as the concentration of treated GNP and GNPs-ALD increased. This effect was especially pronounced in the case of cells treated with GNPs-ALD. In this case, the number of TRAP+MNCs decreased from 26.67 to 15.33 and 2.33 when treated GNPs-ALD at 1 μM, 10 μM, and 20 μM concentrations, respectively.

### Inhibitory effects on osteoclastogenic gene expression

**Figure 4:**
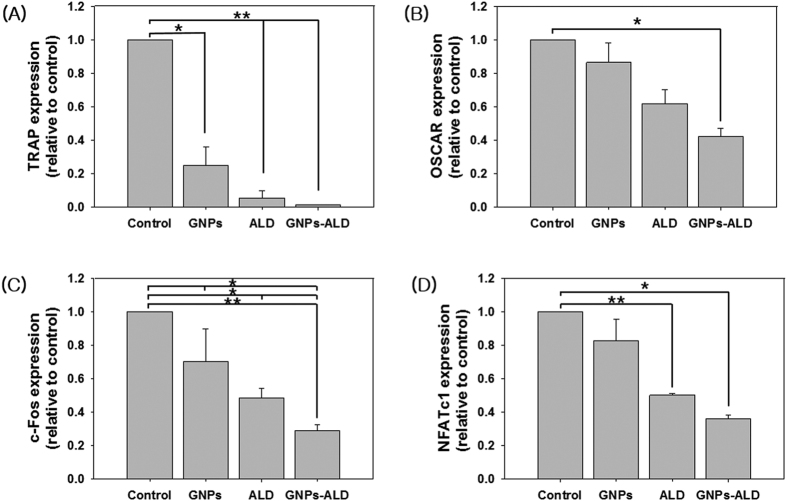
Gene expressions of TRAP (**A**), OSCAR (**B**), c-Fos (**C**) and NFATc1 (**D**) on non-treated media. Also shown with media treated with 20 μM GNPs, 7.32 μg/ml ALD or 20 μM GNPs-ALD media after osteoclast formation in control group. All groups were treated with 30 ng/ml M-CSF and 100 ng/ml RANKL for 5 days. Results are mean ± SD of triplicate experiments: **p < 0.01, *p < 0.05, significant difference as compared with each other.

The genes TRAP, OSCAR, c-Fos, and NFATc1 are known to induce cell differentiation to osteoclasts^26^,^27^,^28^. The inhibitory effects of GNPs, ALD, and GNPs-ALD were evaluated by real-time polymerase chain reaction (RT-PCR) by measuring these osteoclast gene markers (Fig. 4). The negative control group, which had no RANKL, showed nearly no expression of these genes (data not shown). The expression of all markers that were measured in this study were decreased by GNPs, ALD, and GNPs-ALD relative to the positive control. The GNPs-ALD showed a greater inhibitory effect as compared to the other groups. The mRNA expression of TRAP was significantly decreased for all experimental groups (Fig. 4A).

### Histological and relative femur wet weight analysis

**Figure 5:**
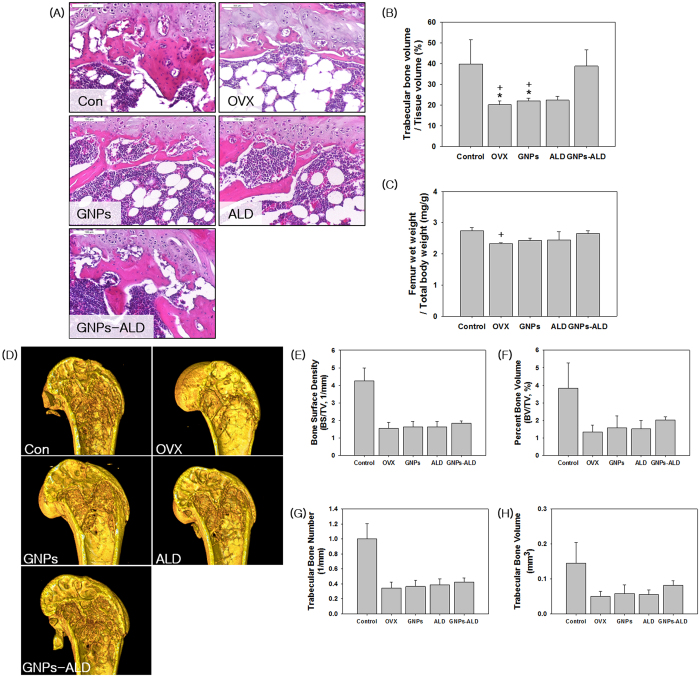
Histological, relative femur wet weight and μ-CT analysis from Control, OVX, GNPs, ALD and GNPs-ALD groups. Sections of trabecular bone in the metaphyseal region of the distal femur were stained using Hematoxylin & Eosin and observed by using a microscope (x100) (**A**). Trabecular dimensions and continuity are greater in Con and GNPs-ALD groups than the other groups. Relative trabecular bone volume showed OVX, GNP, ALD groups had lower trabecular bone volume than Con and GNPs-ALD groups (**B**). Averages of bilateral femur wet weight (mg) per total body weight (g) were calculated (**C**). Relative femur weights of GNPs-ALD group were higher than other groups in the ovariectomized groups (except Con group). Results are mean ± SD of triplicate experiments: ^+^p < 0.05, significant difference as compared with control group and *p < 0.05, significant difference as compared with GNPs-ALD group. Scale bars are 100 μm. Reconstruction images of femur (**D**) and average value of bone surface density (1/mm, **E**), percent bone volume (%, **F**), trabecular bone number (**G**) and trabecular bone volume (**H**) by μ-CT. These were evaluated after producing the femur images as 2240 × 2240 pixel, 5.33 μm resolution and 4 frame averaging.

Figure 5A shows an image of sectioned trabecular bone in the metaphyseal region of the distal femur. The adipose tissue, fibrous tissue, bone tissue and bone marrow cells are displayed as white, pale pink, dark pink and purple, respectively^29^,^30^,^31^. The histomorphometry showed all ovariectomized groups, except GNPs-ALD group had lower volume of trabecular bone than Con and GNPs-ALD groups (Fig. 5A). The ovariectomized mice had a reduction of 17.7% in the trabecular bone volume ratio. However, the value of GNPs-ALD group was 21.03% higher than the OVX group in relative trabecular bone volume analysis. There was no significant difference between Con and GNPs-ALD groups (Fig. 5B). These trends indicating high therapeutic effect of GNPs-ALD similarly appeared in the femur wet weight per total body weight analysis (Fig. 5C).

### The quantification of bone samples by micro-computed tomography (μ-CT)

We carried out an animal model test to demonstrate the therapeutic effect of GNPs, ALD, and GNPs-ALD *in-vivo*. There was no particular change in body weight during the experimental period for all groups (Supplementary Fig. S1). Among various methods of analysis, μ-CT analysis was able to measure the changes of femoral bone in two-dimensional and three-dimensional views. The results of the μ-CT measurements are shown in Fig. 5(D–H). Reconstructed 3D images of distal femur showed that the trabecular pattern of OVX group were relatively vacant as compared to the other groups. On the other hand, GNPs-ALD groups had a more highly maintained trabecular bone volume as compared with GNPs or ALD treated groups. For the quantitative analysis, bone surface density {BS/(tissue volume, TV)}, percent bone volume (BV/TV), trabecular bone number (TBN), and trabecular bone volume (TBV) were also examined. As shown in Fig. 5E–H, the Con group had significantly higher bone formation than the OVX group, which received no treatment. The GNPs, ALD and GNPs-ALD groups displayed higher values for these parameters than the OVX group in all μ-CT analysis. In addition, GNPs alone showed a relatively positive effect when compared with OVX group in measurement of BV/TV and TBV. These increased by 19% and 16%, respectively. The GNPs-ALD groups demonstrated a 17%, 50%, 22% and 64% greater BS/TV, BV/TV, TBN and TBV than the OVX groups, respectively.

## Discussion

Inorganic based nanoparticles have been made of various materials such as carbon nanotubes, silver, graphene, silica, and others. However, many nanoparticles have toxicity towards tissue, cells, and deoxyribonucleic acid (DNA) according to the nanoparticle characteristics. Karlsson *et al*. described that CuZnFe_2_O_4_, ZnO, and CuO nanoparticles caused DNA damage and cytotoxicity. Especially, the CuO nanoparticles have high cytotoxicity and DNA damage effect when compared with other nanoparticles^32^,^33^. Furthermore, in another study, the same materials acted differently depending on the cell type^34^. Conversely, GNPs are well known to have higher biocompatibility and bio-stability as compared with other nanoparticles^35^,^36^. In addition, The GNPs can be used as an MRI contrast agent by their adsorption to bone tissue when injected into the body. They can be applied as an image sensor when combined with a fluorescence dye^37^,^38^. Recent studies have reported that GNPs are effective in promoting osteo-differentiation and inhibiting osteoclast differentiation. Most GNP effects have been observed under *in-vitro* studies and, as such, validation by *in-vivo* experiments are required. Recent research on osteoclasts suggest that GNPs have no cytotoxicity and genotoxicity. Instead, they promote cell proliferation within a certain concentration range^39^. According to previous research, GNPs are bio-accumulated in various internal organs based on their diameter (Heart–5 nm, liver–10 nm, kidney-5 nm, spleen-30 nm). However, GNPs that bio-accumulated in the spleen displayed no toxicity to the body^21^. Thus, in this study, we used spheroidal GNPs with 30 nm diameter. The 30nm GNPs were synthesized and ALD was conjugated onto their surface by mixing with ALD solution. This occurred as the citrate of GNPs were displaced by the primary amines to form partially covalent bonds towards ALD^40^,^41^. The conjugated GNPs were characterized by using a UV-vis spectrophotometer and the result is shown in Fig. 1E. The wavelength curves of GNPs-ALD (531 nm) were shifted to the right by 6 nm as compared to the wavelength curve of GNPs (525 nm). This might be due to the change in the surface plasmon resonance by presence of ALD^42^. This matches well with previous reports, which indicated that the absorbance of GNPs having a diameter of about 30 nm were found to be at a wavelength of 527 nm^43^. Distribution of the particle size can be confirmed by DLS graph (Fig. 1C,D). The synthesized particles were formed in the 20–40 nm size range. ALD coupling to the GNPs surface generated no significant difference for the particle size. The polydispersity values of GNPs and GNPs-ALD were found to be 0.182 ± 0.004 and 0.262 ± 0.008 respectively. This confirms that the particles have a uniform size. Many particles are observed to change their size when combined with materials such as drugs and biomolecules^44^. This is because the particles and materials are bound while maintaining a functional group that each have which requires a linker. However, the binding of GNPs and ALD was accomplished by direct substitution of the citrate groups to ALD which had little effect on particle size^40^,^41^. A strong negative charge of ALD was added due to this bonding, which affects the zeta potential. The formation of a negative charge can help to target the particles for bone treatment. Figure 1F shows the ATR-FTIR spectra indicating the presence of chemical bonds within the materials. Bisphosphonate drugs are generally known to have chemical moieties including P = O, C-N, -OH, and so on. The peaks that appeared in ALD such as hydroxyl group (926 cm^−1^), P = O stretching (1020–1050 cm^−1^) and amide I bond (1650 cm^−1^) also appeared in the GNPs-ALD^45^,^46^,^47^. These peaks did not, however, appear in the uncoated GNPs. This result indicates the successful grafting of ALD onto the GNPs surface. The degree of synthesis of the GNPs-ALD can be seen in Fig. 1G. As shown from these results, the weight percentage of ALD was decreased to about half while heating to 800 °C. Accordingly the amount of ALD that was conjugated on GNPs in the GNPs-ALD is twice the difference of the weight ratio of the residual between GNPs and GNPs-ALD after heating to 800 °C. This indicated a quantity of 7.32 wt. % conjugated ALD to GNPs.

Generally, one of the two chains in bisphosphonate drugs has a hydroxyl group to further enhance its affinity for bone calcium. The other chain acts to suppress bone resorption^48^. We have performed a bone-binding test of GNPs-ALD in order to demonstrate the effect of this combined calcium and hydroxyl group (Fig. 1H–L). As expected, the particles were only found on bones treated with the GNPs-ALD. EDX showed that the GNPs were present by measuring the gold (Au) peak. These results suggest that GNPs-ALD directly influence osteoclasts because they have the capacity to adhere strongly to the bone surface when injected into the body. To compare the endocytosis of GNPs and GNPs-ALD, we have observed up-taken particles in the BMMs by using an optical dark field microscope. These particles were well-absorbed into the BMMs, and the amount of intracellular particles had no significant difference. This result demonstrated that the uptake ability of GNPs was maintained even when coupled with ALD. The GNPs-ALD presented a more even distribution than GNPs in the cells. This is due to the electrical repulsive force caused by the negatively charged particles. Additionally, GNPs displayed no BMMS cytotoxicity over the range of concentrations used in our study (Fig. 3A). ALD, however, did display cytotoxicity at concentrations of more than 150 μM (Supplementary Fig. S3B). Thus, the amount of ALD in the GNPs-ALD solution was limited to under 150 μM in all experiments. Receptor activator of nuclear factor kappa-B ligand (RANKL) is an important factor for osteoclast differentiation and has been reported that the manufacturing of reactive oxygen species (ROS) are induced by the RANK-TRAF6 signal pathway^49^. It has been indicated that ROS plays an important role in differentiation of osteoclasts^50^. GNPs weaken the production of ROS by RANKL and significantly increase expression of glutathione peroxidase-1 (Gpx-1)^21^. Upregulated Gpx-1 leads to a profound suppression of osteoclast formation^51^. Thus we applied ALD, which is widely used to treat osteoporosis, in the GNPs for inhibition of OC differentiation. TRAP, one of the enzymes characteristic of osteoclasts, is involved in the act of dephosphorylation of osteopontin and bone sialoprotein in bone matrix^52^. The TRAP staining tests run using various concentrations of particles and ALD, showed that particles inhibited the differentiation of BMMs. In this experiment, TRAP-positive multinucleated cells were inhibited by GNPs in a dose dependent manner. Furthermore, the effect was amplified when used GNPs-ALD (Fig. 3C–O). Previous studies have explained that, during the differentiation of osteoclast, the PU.1 is involved in the transcription control of osteoclast-specific genes, such as TRAP and OSCAR. This occurs through a combination with other transcription factors, such as c-Fos and NFATc1^53^. Thus, the expressions of TRAP and OSCAR are also likely to be suppressed along with suppression of c-Fos and NFATc1. These genes were found to be suppressed by GNPs and ALD. The differentiation of osteoclast was also inhibited by using GNPs and ALD. Furthermore, when the GNPs and ALD were combined, the inhibitory effect was significantly increased (Fig. 4) indicating a synergistic effect. In the RT PCR analysis, the concentration of ALD was fixed to 7.32 μg/ml. This was selected as it held the concentration of ALD consistent between the ALD group and the GNPs-ALD group, as determined by the TGA results (Fig. 1G). This allowed for direct comparison between these groups.

In the animal model test, OVX mice were divided into four groups. Each group was orally administered water, GNPs, ALD, or GNPs-ALD, respectively. The biocompatibility of GNPs, ALD and GNPs-ALD was evaluated by comparing body weight changes^54^. As shown in supplementary Fig. S1, the weight change patterns were similar for all groups. This indicates that the administered materials had little or no toxicity. In μ-CT analysis, the BS/TV, BV/TV, TBN and TBV are used as the ordinary parameters of bone tissue^55^,^56^, and these have been used to characterize bone porosity in many studies. The measured BS/TV, BV/TV, TBN and TBV values of OVX was significantly lower than Con. This showed that the ovariectomy procedure successfully induced osteoporosis in normal mice. As shown by histological and relative femur wet weight analysis, the quantity of trabecular bone in GNPs and ALD groups were similar or lower than the OVX group. However, the GNPs-ALD group displayed a higher value for trabecular bone than GNPs and ALD groups. These results show that the therapy effect of GNPs-ALD was higher than GNPs or ALD. This is suspected to be due to the enhanced cell uptake of ALD as increased by combining it with GNPs. In addition, GNPs were targeted to the bone tissue rather than being absorbed to other tissues or being discharged from the body. These results indicate the potential for increasing the effective treatment of OP.

In conclusion, we prepared gold-nanoparticles conjugated to alendronate, which can inhibit the differentiation of BMMs to osteoclasts. The composite particles were characterized using various techniques such as UV-Vis spectroscopy, TEM, SEM, EDS, ATR-FTIR, DLS and TGA. The inhibition effects of the formed particles were confirmed by TRAP activity, TRAP staining, RT PCR, and animal test. These exploratory results indicate that GNPs-ALD have a synergistic effect of suppressing differentiation more than GNPs and ALD alone. Moreover, conjugated particles were found to have no cytotoxicity at the concentrations tested as confirmed by WST assay. In conclusion, these results show that GNPs-ALD has the highest inhibitory effects towards osteoclast differentiation of bone marrow-derived macrophage. Thus, GNPs-ALD can play a key role as useful agents for osteoporosis therapy.

## Methods

### Materials

Gold (III) chloride hydrate (99.999% trace metals basis), sodium citrate, red blood cell lysing buffer, and acid phosphatase, leukocyte acid phosphatase (TRAP) kits were purchased from Sigma-Aldrich (St Louls, MO). TRACP&ALP Assay kit was purchased from Takara (Seoul, Korea). Alendronate sodium trihydrate was purchased from TCI (Tokyo Chemical Industry Co., LTD, Japan). Macrophage colony stimulating factor (M-CSF) and recombinant murine sRANK ligand were purchased from Peprotech Korea (Seoul, Korea). RIPA lysis buffer and PBS 10X were purchased from Millipore. Protease inhibitor cocktail tablets were purchased from Roche Diagnostics Indianapolis (USA). ICR six-week old male mice, used for extraction of BMMs, were purchased from Young bio (Sung-nam, Korea). Minimum Essential Media (MEM) Alpha Medium, fetal bovine serum (FBS), antibiotic agents (penicillin/streptomycin, PS) and Dulbecco’s Phosphate Buffered Saline (DPBS) were purchased from GIBCO BRL (Invitrogen Co., USA). 48-well cell culture plates and cell culture dishes (100 mm × 20 mm) were purchased from Corning Incorporated (New York, USA). EZ-Cytox (enhanced cell viability assay kit) was purchased from Dogen (Seoul, Korea).

### Equipment

FE-SEM and EDX observations were carried out using a Field Emission S-4700 (Hitachi High Technologies Corp., Tokyo, Japan). UV-vis was measured using an UV-1650PC (Shimadzu, Japan) spectrophotometer. Thermal analyses such as differential scanning calorimetry (DSC) and TGA were performed using a SDT Q600 (TA instrument, USA). ATR-FTIR measurements were carried out using an IFS66V/S & HYPERION 3000 (Bruker Optiks, Germany). DLS was performed using a 90 Plus Particle Size Analyzer (Brookhaven Instruments Corporation, NY, USA). Light scattering image was obtained using a Kodak Image Station 4000 MM (Digital Imaging Systems, New Haven, CT, USA). All equipment were operated by the Central Laboratory for Instrumental Analysis of Kyunghee University (Seoul, Korea), Center for Research Facilities Kyunghee University (Yongin, Korea) and Korea Institute of Science and Technology (Seoul, Korea).

### Preparation of GNPs and GNPs-ALD

GNPs were produced using the citrate reduction of HAuCl_4_ as previously described^57^. Briefly, 0.02% HAuCl_4_ solution was refluxed at 100 °C, and then 2% sodium citrate solution was added rapidly. After the solution changed color to dark red, the solution temperature was maintained for 15 min and then cooled slowly to room temperature.

The GNPs were conjugated to ALD by addition of 0.204 mM alendronate sodium trihydrate solution and stirring for 48 h at room temperature. After that, the mixtures were dialyzed (MWCO : 3500 Da) for four days to eliminate unattached ALD. GNPs and GNPs-ALD solutions were kept refrigerated (4 °C) and were centrifuged at 13000 rpm for 10 minutes to form a pellet of GNPs and GNPs-ALD. This pellet was for use in characterization and further experiments. Moreover, these pellets were dehydrated by freeze drying in a TFD series (Ilshinbiobase, Korea) apparatus for ingredient analysis.

### Characterization

For UV-vis spectrum analysis, the concentration of GNPs solution was adjusted to be equal with the concentration of GNPs-ALD solution. This was necessary as the concentration of GNPs-ALD was decreased during dialysis after synthesis of the two materials in the GNPs-ALD production process. The absorption spectra of these solutions were measured using a UV-1650PC. These solutions were lyophilized prior to measuring TGA and ATR-FTIR. TGA analysis was performed under a high purity nitrogen flow of 100 ml/min. The temperature of the furnace was set to increase at 10 °C/min and up to a maximum of 800 °C. DLS and zeta potential analysis were performed to determine the surface charge and size of nanoparticles at a concentration of 270 μM at 25 °C.

### Evaluation of particles attachment to bone surface

Extracted tibias and femurs, after being used for isolation of BMMs, were used for these tests. These bones were immersed in DPBS, GNPs and GNPs-ALD solutions, respectively, with shaking at 37 °C. After 24 hours, the tibias and femurs were washed in DPBS by ultra-sonication for 10 minutes and were dehydrated by freeze-drying for 24 hours. For FE-SEM and EDX measurement, these samples were coated by platinum in the vacuum chamber, and then were observed using Field Emission S-4700.

### Isolation and cultivation of BMMs

BMMs were isolated from bone marrow in tibias and femurs of 6 week-old ICR mouse (Young bio, Korea). Mice were sacrificed by cervical vertebral dislocation and then the hind legs of the mouse were severed from the trunk. Bone marrow was isolated from the legs by rinsing the bone marrow cavity with MEM alpha medium using a 1-mL syringe and 25-gauge needle. Then BMMs were isolated from the mixture of bone marrow and MEM alpha medium. BMMs were primary cultured in a petri dish filled growth medium (MEM alpha medium containing 1% PS, 10% FBS and 30 ng/ml M-CSF) at 37 °C in a humidified 5% CO_2_ incubator. After BMMs were cultured for four days, cells were scraped from the petri dish using a cell scrapper and were seeded in the well culture plates. In order to differentiate to osteoclasts, 100 ng/ml RANKL was added to the growth mediums (ODM). After seeding the BMMs, the medium was replaced every three days.

### Optical dark field analysis

The isolated BMMs were seeded in a 13Ø mm hole confocal dish (SPL, Korea) at a density of 5 × 10^5^ cells per dish filled with growth medium. After 24 hours, the growth medium was changed to a new medium containing 20 μM of GNPs or GNPs-ALD, and incubated at 37 °C in a humidified 5% CO_2_ for 12 hours. The BMMs that endocytosed GNPs or GNPs-ALD were washed with DPBS, and then these were immobilized with 3.7% formaldehyde (Sigma-Aldrich, St Louis, MO) at room temperature for 20 min. The fixed BMMs were rinsed out using DPBS. The endocytosed particles were visualized using a 12-bit charge coupled device camera equipped with a special C-mount lens. To carry out the quantitative analysis, the obtained images were evaluated by Image J program (Total white spots ratio in the whole area, %).

### Cytotoxicity test of BMMs

BMMs were cultured in growth medium for 4 days and were seeded in 48 well culture plates at a density of 1 × 10^4^ cells per well. After 24 hours of seeding, growth mediums were changed to new medium containing varying concentrations of GNPs and ALD. The viabilities of BMMs treated by GNPs and ALD were evaluated by using the EZ-Cytox and were checked at 1 day, 2 days, and 5 days. After 1 day, 2 days, and 5 days of treatment, each group was treated with MEM alpha medium containing 10% EZ-Cytox, and were measured using an ELISA reader at 450 nm wavelength^58^.

### TRAP staining

Anti-osteoclastic differentiation effects of GNPs, ALD, and GNPs-ALD on BMMs were quantitatively and qualitatively evaluated using TRAP staining kits. BMMs were seeded in 48 well culture plates at a density of 2 × 10^4^ cells per well. The cells were cultured using ODM until osteoclasts were observed by microscopy (about five days). In order to measure differentiation, the cells were fixed with 4% formalin and were washed using saline. Subsequently, the cells were treated by staining kit. TRAP staining was observed by microscopy, and the number of TRAP-positive multinucleated cells (TRAP+MNCs) was counted.

### Real-time PCR

The BMMs were cultured at a density of 2 × 10^4^ cells per well and treated with 20 μM GNPs, GNPs-ALD, and 7.32 μg/ml ALD, for five days. The RNA of the cells was isolated by RNeasy Plus Mini Kit (Qiagen, CA, USA). The cDNA was synthesized using the AccuPower CycleScript RT Premix (Bioneer, Daejeon, Republic Korea). The primers of the measured mRNA genes were as follows: TRAP–5′-CTG GAG TGC ACG ATG CCA GCG ACA-3′ (sense) and 5′-TCC GTG CTC GGC GAT GGA CCA GA-3′ (antisense), osteoclast-associated receptor (OSCAR)–5′- CTG GTA ACG GAT CAG CTC CCC AGA-3′ (sense) and 5′- CCA AGG AGC CAG AAC CTT CGA AAC T-3′ (antisense), c-Fos–5′-CTG GTG CAG CCC ACT CTG GTC -3′ (sense) and 5′- CTT TCA GCA GAT TGG CAA TCT C -3′ (antisense), NFATc1–5′-CTC GAA AGA CAG CAC TGG AGC AT-3′ (sense) and 5′-CGG CTG CCT TCC GTC TCA TAG-3′ (antisense), and glyceraldehyde 3-phosphate dehydrogenase (GAPDH)–5′- CAT GGC CTT CCG TGT TCC TAC CC -3′ (sense) and 5′- CCT CAG TGT AGC CCA AGA TGC CCT-3′ (antisense). The gene expression was evaluated by using iQ SYBR Green supermix (Bio-Rad Hercules, CA, USA). The control group treated by medium including RANKL and M-CSF was set at 1 fold and the experimental groups were compared to the control group. The RT PCR amplifications were carried out for 20 seconds at 95 °C, 30 seconds at 60 °C, and 20 seconds at 72 °C for 40 cycles after the initial denaturation step of 10 minutes at 95 °C. Threshold cycle values were calculated by using a comparative cycle threshold (CT) method. All results were standardized by GAPDH.

### Mouse models

Female 10-week-old C57B1/6 mice (*n* = 40, average weight = 20 g) were purchased from Orient Bio (Seongnam, Korea). After 2 weeks, the OVX group mice (*n* = 32) had their ovaries removed and the control group (Con) mice (*n* = 8) had a sham surgery performed in which their ovaries were not removed. The ovariectomized mice were divided into four groups (*n* = 8 per group) as OVX (control, given water), GNPs (500 μM of gold nanoparticles), ALD (50 μM of alendronate) and GNPs-ALD (500 μM gold nanoparticles conjugated 50 μM alendronate). Each solution (0.5 mL) was administered orally to the experimental groups every day for 12 weeks. All animal tests and care were approved by the Institutional Animal Care and Use Committee of Konkuk University (KU14112), and the methods were carried out in accordance with approved guidelines and regulations.

### Histological analysis

After μ-CT analysis, for histological analysis, the isolated femora were prepared for hematoxylin and eosin (H&E) staining. The specimens were fixed using 10% neutralized buffered formalin solution at 4 °C for 24 h, and were embedded in paraffin. After 4 μm-thick sections were stained with H&E, these were evaluated by microscopy.

### Microcomputed tomography (μ-CT) analysis

The femora enucleated from mice of all experimental groups were examined by μ-CT. These examinations were used to determine the change in the trabecular bone and were carried out by using a SkyScan1173 (SKYSCAN, Belgium) at 5.33 μm resolution. The degree of bone density based on μ-CT was quantified by using the mean gray value and standard deviation of the region of interest (ROI). Morphometric parameters including bone surface density (bone surface/tissue volume, 1/mm), percent bone volume (bone volume/tissue volume, %), trabecular bone number (1/mm) and trabecular bone volume (mm^3^) were obtained by using On-demand software (CyberMed Inc., Korea).

### Statistical analysis

For cell cytotoxicity, TRAP staining, and osteoclast differentiation assays, a quantity of BMMs were seeded in four wells per group for the Con, GNPs, ALD, and GNPs-ALD groups, respectively. For these tests the number of replicates was four. In addition, statistical analyses were performed using analysis of variance (ANOVA) with post-hoc multiple comparisons. For these, the data were tested for normality and homogeneity of variance in advance. In the TRAP staining analysis, only the difference between GNPs and GNPs-ALD was tested using Student’s t-test. All values were expressed as means ± standard deviations and all the experimental groups were compared with the control group.

## Additional Information

**How to cite this article**: Lee, D. *et al*. Inhibition of Osteoclast Differentiation and Bone Resorption by Bisphosphonate-conjugated Gold Nanoparticles. *Sci. Rep.* **6**, 27336; doi: 10.1038/srep27336 (2016).

## Supplementary Material

Supplementary Information

## Figures and Tables

**Table 1:** Mean diameter, polydispersity measured by dynamic light scattering and zeta potential for GNPs and GNPs-ALD.

	**Mean diameter (nm)**	**Polydispersity (PDI)**	**Zeta potential (mV)**
GNPs	34.7 ± 1.8	0.182 ± 0.004	0
GNPs-ALD	32.9 ± 0.7	0.262 ± 0.008	−40.75 ± 2.71

Data are provided as mean ± standard deviation (*n* = 3).
