# Supplementary material for: Inhibition of Osteoclast Differentiation and Bone Resorption by Bisphosphonate-conjugated Gold Nanoparticles
Source: Sci Rep. 2016 Jun 2;6:27336. doi: 10.1038/srep27336 (PMC4890291; doi:10.1038/srep27336)
Supplement: Supplementary Information [file srep27336-s1.pdf]

Inhibition of Osteoclast Differentiation and Bone Resorption by Bisphosphonate-conjugated Gold Nanoparticles

Donghyun Lee[1,+], Dong Nyoung Heo[3,+], Han-Jun Kim[4], Wan-Kyu Ko[1], Sang Jin Lee[1], Min Heo[1], Jae Beum Bang[5], Jung Bok Lee[6], Deok-Sang Hwang[7], Sun Hee Do[4,*] and Il Keun Kwon[2,*]

[1]Kyung Hee University, Department of Dentistry, Graduate School, 26, Kyungheedae-ro, Dongdaemun-gu, Seoul 02477, Korea

[2]Kyung Hee University, Department of Dental Materials, School of Dentistry, 26, Kyungheedae-ro, Dongdaemun-gu, Seoul 02477, Korea

[3]The George Washington University, Department of Mechanical and Aerospace Engineering, Washington DC 20052, United states

[4]Konkuk University, Department of Clinical Pathology, College of Veterinary Medicine, Seoul 05029, Korea

[5]Kyung Hee University, Department of Dental Education, School of Dentistry, 26, Kyungheedae-ro, Dongdaemun-gu, Seoul 02477, Korea

[6]Vanderbilt University, Department of Biomedical Engineering, Nashville, TN 37235, United States

[7]Department of Korean Gynecology, Conmaul Hospital, Kyung Hee University, Seoul 02477, Korea

*Correspondence and requests for materials should be addressed to S.H. (email: shdo@konkuk.ac.kr) and I. K. (email: kwoni@khu.ac.kr)

[+]These authors contributed equally to this work.

Supplementary Figure captions

Supplementary Figure S1. Body weight changes in control mouse (Con), OVX mouse receiving no treatment (OVX), gold nanoparticles (GNPs), alendronate (ALD) and alendronate-conjugated gold nanoparticles (GNPs-ALD) for 12 weeks. Each point represents the mean ± SD, error bars in the figure are presented as SD, n=8.

Supplementary Figure S2. Micro-computed tomography (μ-CT) image of Con (A), OVX (B), GNPs (C), ALD (D) and GNPs-ALD (C).

Supplementary Figure S3. Evaluation of cytotoxicity of BMM cells on high concentrations of GNPs (A) and ALD (B). Results are mean ±SD of triplicate experiments.

Supplementary Figures

S1.

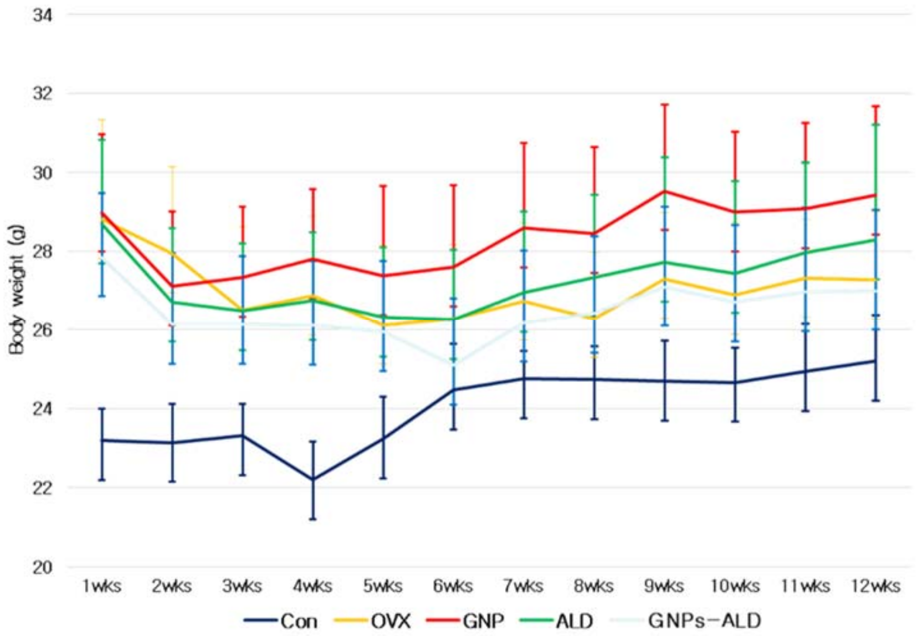

S2.

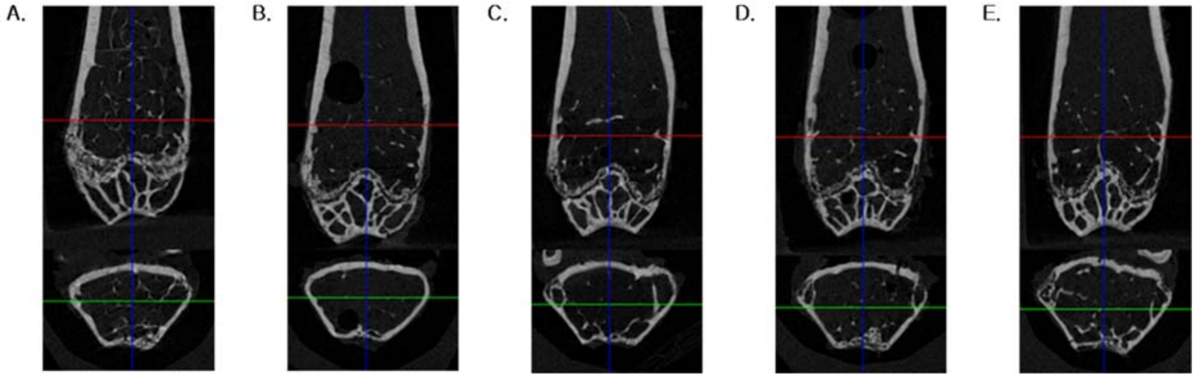
A.
B.
C.
D.
E.

S3.

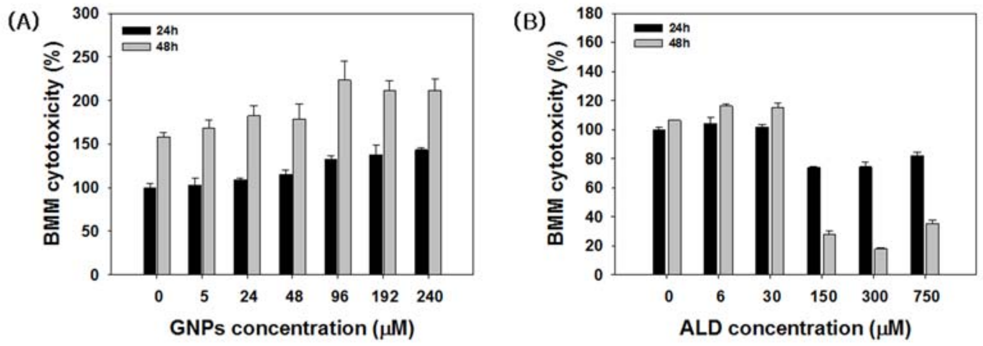
(A)
24h
48h
BMM cytotoxicity (%)
300
250
200
150
100
50
0
0
5
24
48
96
192
240
GNPs concentration (μM)
(B)
24h
48h
BMM cytotoxicity (%)
180
160
140
120
100
80
60
40
20
0
0
6
30
150
300
750
ALD concentration (μM)
